# The characteristics of krill swarms in relation to aggregating Antarctic blue whales

**DOI:** 10.1038/s41598-019-52792-4

**Published:** 2019-11-11

**Authors:** E. J. Miller, J. M. Potts, M. J. Cox, B. S. Miller, S. Calderan, R. Leaper, P. A. Olson, R. L. O’Driscoll, M. C. Double

**Affiliations:** 1 Australian Antarctic Division, 203 Channel Highway, Kingston, Tasmania Australia; 2 E Miller Consulting, Hobart, Tasmania Australia; 3 The Analytical Edge, PO Box 47, Blackmans Bay, Tasmania Australia; 4 Scottish Association for Marine Science, University of the Highlands and Islands, Oban, Argyll UK; 5 International Fund for Animal Welfare, 87-90 Albert Embankment, Lambeth, London UK; 6 Southwest Fisheries Science Center, National Marine Fisheries Service/National Oceanic and Atmospheric Administration, La Jolla, California USA; 7 National Institute of Water & Atmospheric Research Limited, Wellington, New Zealand

**Keywords:** Ecosystem ecology, Ecosystem ecology

## Abstract

We model the presence of rare Antarctic blue whales (*Balaenoptera musculus intermedia*) in relation to the swarm characteristics of their main prey species, Antarctic krill (*Euphausia superba*). A combination of visual observations and recent advances in passive acoustic technology were used to locate Antarctic blue whales, whilst simultaneously using active underwater acoustics to characterise the distribution, size, depth, composition and density of krill swarms. Krill swarm characteristics and blue whale presence were examined at a range of spatiotemporal scales to investigate sub meso-scale (i.e., <100 km) foraging behaviour. Results suggest that at all scales, Antarctic blue whales are more likely to be detected within the vicinity of krill swarms with a higher density of krill, those found shallower in the water column, and those of greater vertical height. These findings support hypotheses that as lunge-feeders of extreme size, Antarctic blue whales target shallow, dense krill swarms to maximise their energy intake. As both Antarctic krill and blue whales play a key role in the Southern Ocean ecosystem, the nature of their predator-prey dynamics is an important consideration, not only for the recovery of this endangered species in a changing environment, but for the future management of Antarctic krill fisheries.

## Introduction

Describing the drivers behind animal distribution is fundamental in understanding their ecology, and key to effective conservation and management in predicting how animals will respond to environmental change. Prey availability is assumed to be a major driver of habitat selection for animals with high energetic needs. As the largest animals to have ever lived, Antarctic blue whales require large amounts of food, and as lunge feeders, they expend substantial amounts of energy in capturing prey^1–3^. The Southern Ocean supports an extraordinary number of predators, including whales, seals, penguins, seabirds and fish, each with their own foraging strategies and abilities. While the foraging niche of these species varies greatly^4,5^, most are reliant on Antarctic krill (*Euphausia superba*), the critical link in the Southern Ocean food web^6^.

Krill distribution is highly variable throughout the Southern Ocean ranging from large dispersed patches^7^ to dense and discrete swarms^8,9^. Aggregations vary in horizonal length (tens to thousands of metres), vertical height (tens of meters) and numerical density of krill (<1 to 1000’s of individuals m^−3^)^8^. Swarms may be made up of varying sizes of krill, and the depth of swarms and distance between them is also dynamic. All of these characteristics influence the detectability, availability and energy quality of prey.

Until recently, studies of predator-prey interactions in the Southern Ocean largely focussed on land-based krill predators^10–13^. Many baleen whales migrate to Antarctic feeding grounds in the summer where they feed primarily on Antarctic krill^14–17^. During this time, the fine-scale distribution of these whales is assumed to be highly driven by the distribution and availability of krill^18–20^. Townsend^21^ was one of the first to suggest a direct association between cetacean distribution and prey availability, which would require that cetaceans have knowledge of, or are able to predict prey distribution, and that the prey are accessible. The scale-dependency of predator-prey relationships varies with prey predictability and patchiness^20,22^.

Studying the use of habitat by cetaceans presents several challenges. They often live in remote, inaccessible environments, can undertake long migrations, and spend the majority of their time underwater. Few studies have focused on the relationship between baleen whales and krill in the Antarctic, and most behavioural studies to date have taken place around the Antarctic Peninsula on humpback whales (*Megaptera novaeangliae*), minke whales (*Balaenoptera bonaerensis*), and fin whales (*Balaenoptera physalus*) over a variety of spatial scales (1–1000 s of kms)^23–28^. A variety of methods have been used in these studies to assess whale presence and behaviour, including visual surveys^25,26,29^, suction tags^23,24,27^ and satellite tags^28^. Those that collected concurrent data on krill distribution did so using either active acoustics (scientific echosounders), allowing finer spatial sampling of individual swarms^24,26,29^ or net hauls, resulting in coarser spatial sampling but direct measurements and identification of krill^25,26^. Other studies did not collect data on krill directly, but tracked whale lunges to assess feeding behaviour^23,27^.

From these studies, several associations have been found between whales and krill around the Antarctic Peninsula, including spatial clustering with distinct hotspots at macro- and meso-scales^24,25,28^. At smaller scales, fin and humpback whales were associated with moderate^26^ and high^24^ levels of krill biomass respectively. Whales were also associated with krill at shallower depths^24,30^ and vertical resource partitioning was apparent, with humpback whales associated with shallower krill than minke whales^29^. There was also some evidence of size-selectivity, with humpback, minke and fin whales feeding on progressively larger krill^25^. One study at South Georgia^31^ found a positive relationship between whale abundance and mean krill biomass at meso-scales (80 × 100 km) which weakened at smaller scales due to a more frequent absence of whales in areas of high krill biomass, primarily inshore. This study suggests that biomass may be too simplistic a measure at fine resolutions, and that detailed data on krill swarm structure and density are required to thoroughly assess krill availability.

Little is known about the foraging habits of Antarctic blue whales, despite their extreme biology and past exploitation. The number of Antarctic blue whales was reduced to <1% of their estimated pre-whaling abundance of 239,000 (95% CI: 202,000–311 000)^32,33^. These animals are challenging to study not only due to their rarity, but also their wide-ranging distribution, spread out throughout the Southern Ocean during the summer months^33^. Only a single recent study, conducted outside of the summer feeding season (April-May) near the Western Antarctic Peninsula, has focussed on the relationship between Antarctic blue whales and krill^34^. That study found a negative association between blue whale call presence and krill biomass in the top 100 m, using passive acoustics to detect, but not localise, calling whales. Despite this limited data collection, there have been several attempts to model the relationship between blue whales and krill^35,36^, and it is believed that this relationship plays an important role in the Southern Ocean ecosystem^37^. Both krill and whales act as ‘ecosystem engineers’, enhancing primary productivity through nutrient recycling^36–38^. Models have suggested positive feedback between the population abundance of whales and krill^36^ and that an expanding krill fishery could have a negative impact on the recovery of blue whales^35^.

By employing recent advances in passive acoustic technology it is now possible to locate Antarctic blue whales reliably and efficiently^39^ using their loud and distinctive vocalisations. Recent studies in the Southern Ocean have combined real-time passive acoustic tracking with visual observations, allowing adaptive surveying of Antarctic blue whales over both large and small spatiotemporal scales^39,40^. This provides opportunities to characterise associated ecological and environmental data, including krill, oceanography and ice, and facilitates localised studies of the whales’ feeding ecology.

A key component of the multidisciplinary New Zealand-Australia Antarctic Ecosystem Voyage (January 29^th^ – March 11^th^, 2015) was a survey of Antarctic blue whales and krill carried out as part of the Antarctic Blue Whale Project of the International Whaling Commission’s Southern Ocean Research Partnership (IWC-SORP). The goal of this research was to use passive acoustics and visual observations to localise groups of vocalising and surfacing Antarctic blue whales, and concurrently use active acoustics to map the distribution and characteristics of krill swarms within their vicinity. Our motivation for conducting this analysis was to determine the utility of combining these methods for sub meso-scale prey field mapping around Antarctic blue whales. Active acoustics is a proven, mature technology and is capable of observing krill in an undisturbed form^38,41^. For the purposes of this voyage, active acoustics enabled observations of krill enroute to blue whale feeding grounds, as well as more localised surveys of krill swarms within a blue whale aggregation. The aim of the analysis presented here was to compare the characteristics of krill swarms within the vicinity of Antarctic blue whales to those demonstrably far from groups of blue whales.

## Results

**Figure 1:**
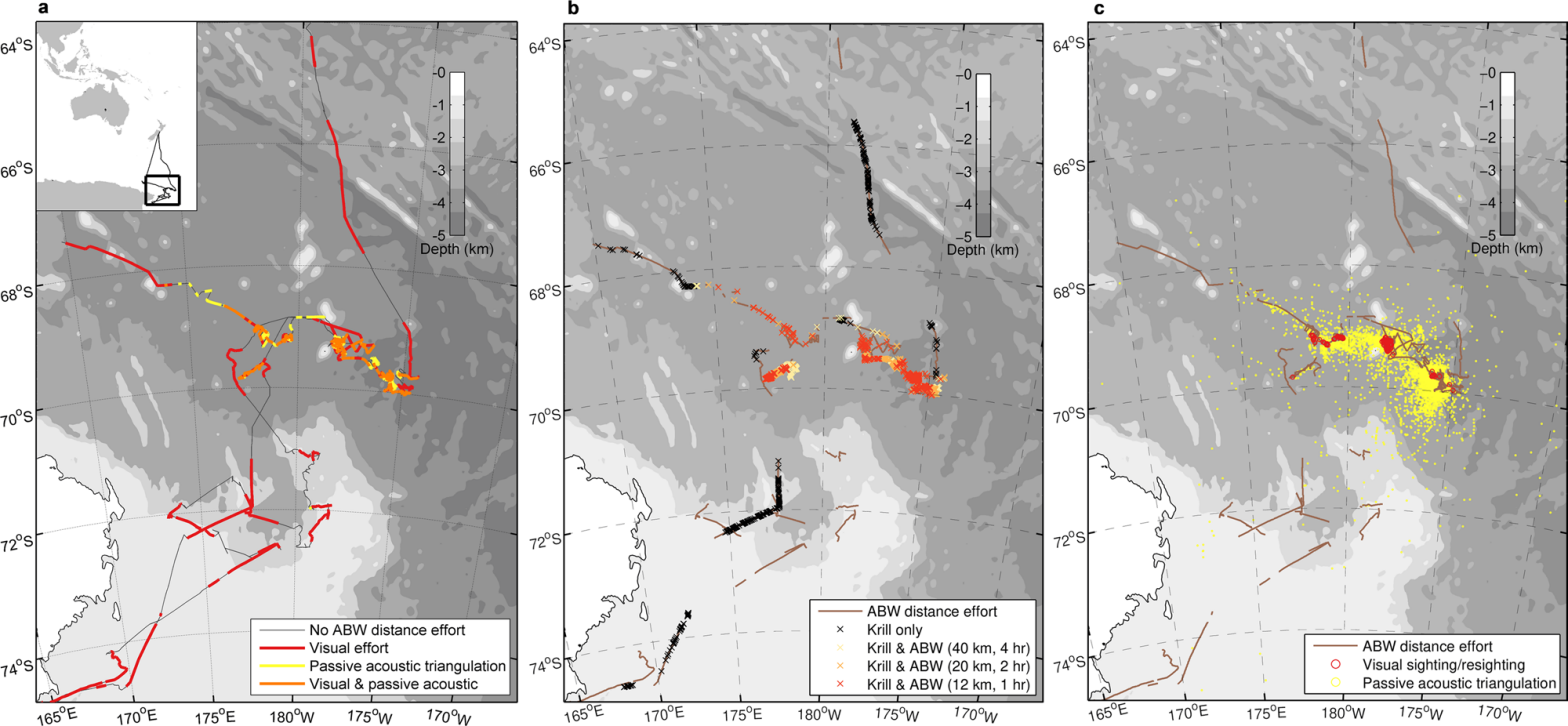
(**a**) Ship’s track showing blue whale survey effort during active acoustic data collection of krill swarms in the Ross Sea region. Thin black line indicates times when there was no effort for measuring distances to whales. Red line indicates visual observation effort; yellow line indicates ship’s track during passive acoustic triangulation effort (two sonobuoys were deployed simultaneously). Orange line indicates concurrent visual and passive acoustic triangulation effort. (**b**) Crosses indicate the locations of krill swarms detected during blue whale survey effort (brown line). Colours indicate the most proximate distance & timespan for which whales were present. (**c**) Locations of blue whale visual sightings/resightings and passive acoustic triangulations.

The spatial distribution of vocalising blue whales was highly concentrated into aggregations which could be heard from hundreds of kilometres away (Fig. 1). In general, the whales were found close to the ice edge (see Fig. S1, Supplementary Material). A total of 34 sightings and 161 re-sightings of blue whales were made throughout 397 hours of visual sighting effort (Fig. 1).

A total of 310 sonobuoys were deployed throughout the voyage providing over 520 hours of passive acoustic recordings containing 42,489 detections of blue whale calls (tonal song calls or d-calls). A total of 222 hours of listening effort was obtained with two sonobuoys deployed simultaneously, providing 7437 triangulated positions to vocalising blue whales (Fig. 1). Both tonal and frequency modulated d-calls from Antarctic blue whales were detected within and around vocal aggregations.

**Figure 2:**
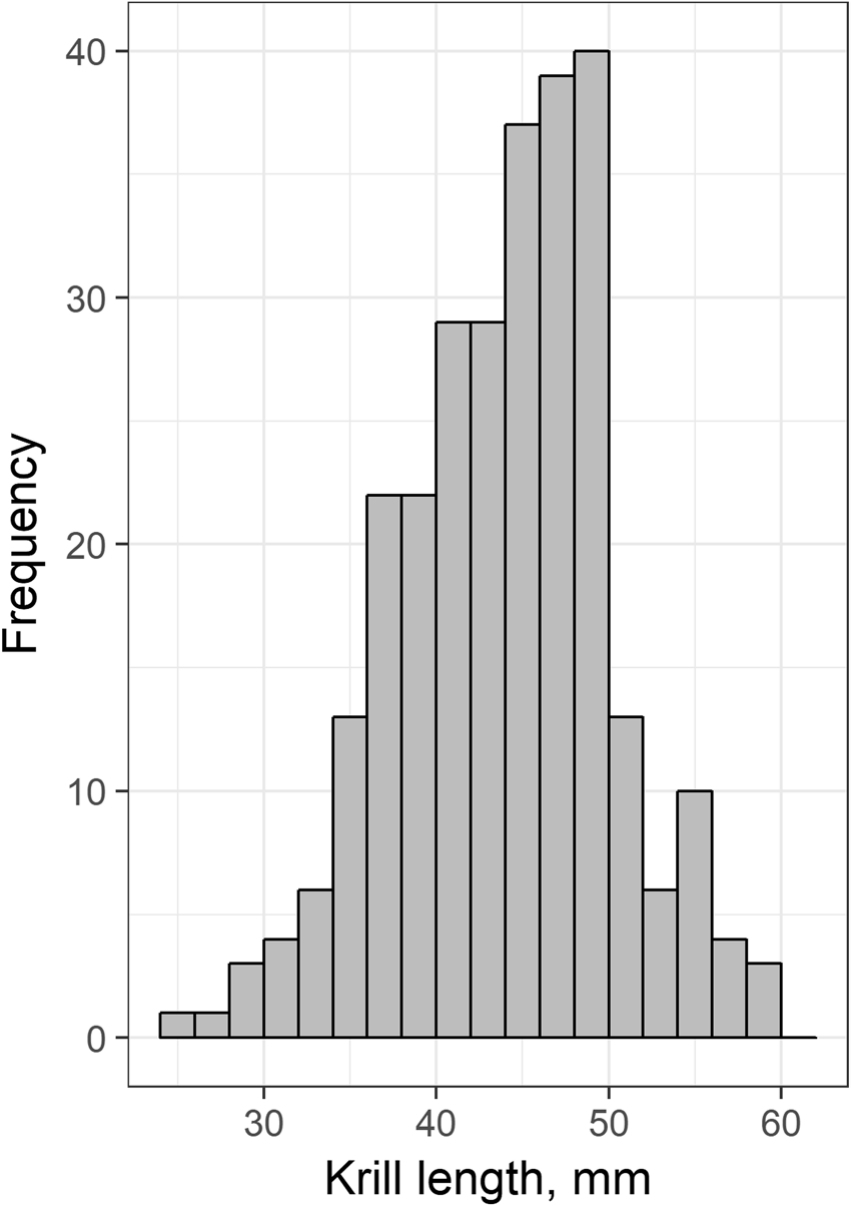
Length-frequency of Antarctic krill (*Euphausia superba*) caught in targeted midwater trawls.

Antarctic krill (*Euphausia superba*) caught in targeted tows ranged from between 26 to 60 mm in length (mean = 44.5 ± 6.2 mm; Fig. 2). Forty krill swarms were detected during periods of high variability in the ship’s heading and subsequently removed from the analysis. Excluding these swarms, a total of 1688 krill swarms were detected along the survey track. The total numbers of swarms detected during periods of visual sighting/passive acoustic effort to locate Antarctic blue whales are presented in Table 1.

**Table 1:** The number of krill swarms detected during visual sighting and/or passive acoustic effort to locate Antarctic blue whales. Forty krill swarms detected during periods of high variability in the ship’s heading were removed from the dataset.

Whale search effort	Number of krill swarms detected
No effort	559
Visual sighting effort only	667
Passive acoustic triangulation effort only	125
Both visual and PA effort	337

**Figure 3:**
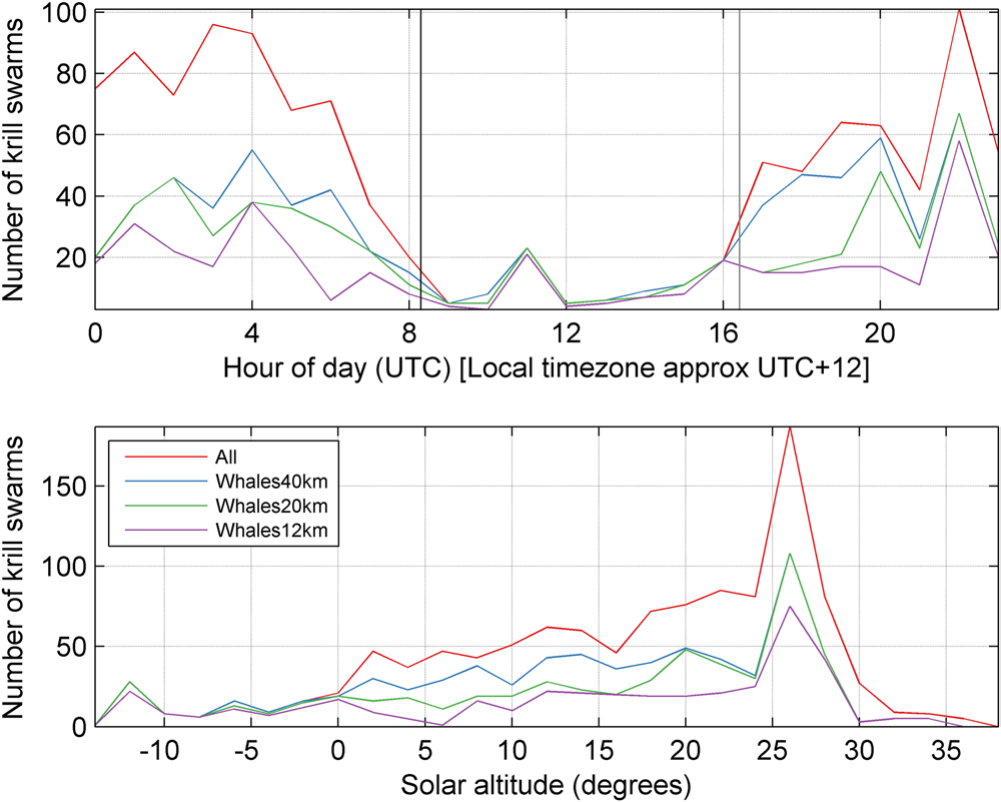
The number of krill swarms detected during periods of whale search effort for each spatiotemporal scale as a function of time of day and solar altitude. Whales were classified as ‘present’ if detected within 12 km/1 hr, 20 km/2 hr, or 40 km/4 hr of a krill swarm using visual sightings and/or passive acoustic triangulation. Solar altitudes  < 0° indicate night time, while solar altitudes > 0° indicate daylight hours. The altitudes that corresponded to solar midnight and solar noon were approximately −12 and 37 degrees respectively, though these changed slightly throughout the voyage depending on latitude and longitude, and day.

Overall, 1129 krill swarms were detected during some form of whale effort (either visual sighting effort or passive acoustic effort, or both; Fig. 1) and therefore were included in our BRT analysis. The 11 measured characteristics of these krill swarms are summarised in Table 2. Of these 1129 krill swarms, the number of swarms detected in the presence and absence of blue whales for each of the three spatiotemporal thresholds examined are shown in Table 3. Krill swarms were detected at all hours of the day, though the short nights during Antarctic summer resulted in our analysis including considerably fewer swarms detected at night (Table 3; Fig. 3). In addition, our ability to detect whales at night was limited to passive acoustics and localisation was only attempted when the sonobuoys indicated whales were in the vicinity of the vessel.

**Table 2:** Summary statistics for the explanatory variables describing krill swarms that were used in the boosted regression tree model. ^a^Beam corrections after Diner^59^. ^b^See Lawson *et al*.^69^ for definition. ^c^See Maclennan *et al.*^60^ for definition of acoustic units.

Explanatory variable	Description	Mean, range, (upper 99.9% quantile)
Corrected area^a,b^	Swarm area corrected for transducer beam shape, m^2^	1636, 19 to 37043 (22576)
Corrected length^a,b^	Swarm length corrected for transducer beam shape, m	146, 4 to 2584 (1081)
Corrected perimeter^a,b^	Swarm perimeter corrected for transducer beam shape, m	448, 25 to 7434 (4140)
Roughness	Roughness, swarm corrected perimeter/swarm corrected area, m^−1^	1, 0 to 11 (2)
Mean depth	Krill swarm mean depth, m	64, 7 to 248 (247)
Mean height	Mean height, i.e. difference between the deepest and shallowest depths of a swarm, m	11, 2 to 79 (44)
dB difference^a^	${\mathrm{MVBS}}_{120kHz}$ - ${\mathrm{MVBS}}_{38kHz}$, dB re 1 m^−1.^ Swarms comprised of larger krill will have lower dB differences	9, 1 to 14 (14)
Biomass density (wet-weight)	Krill swarm biomass density (wet-weight)g m^−3^	122, 2 to 1725 (970)
Swarm backscattering coefficient^c^	Measure of acoustic energy proportional to swarm length, m	4, 0 to 323 (117)
Nearest neighbour distance	Swarm nearest neighbour distance, m	923, 0 to 23618 (16223)
Nearest neighbour depth	Swarm nearest neighbour depth difference, m	33, 0 to 240 (230)

**Table 3:** The number of krill swarms detected during day and night whale search effort in the presence and absence of blue whales for each spatiotemporal scale. Whales were classified as ‘present’ if detected within 12 km/1 hr, 20 km/2 hr, or 40 km/4 hr of a krill swarm using visual sightings and/or passive acoustic triangulation.

Spatiotemporal scale	Number of krill swarms with whales present			Number of krill swarms with whales absent		
	Total	Day	Night	Total	Day	Night
12 km/1 hr	402	335	67	727	710	17
20 km/2 hr	564	485	79	565	560	5
40 km/4 hr	702	618	84	427	427	0

**Figure 4:**
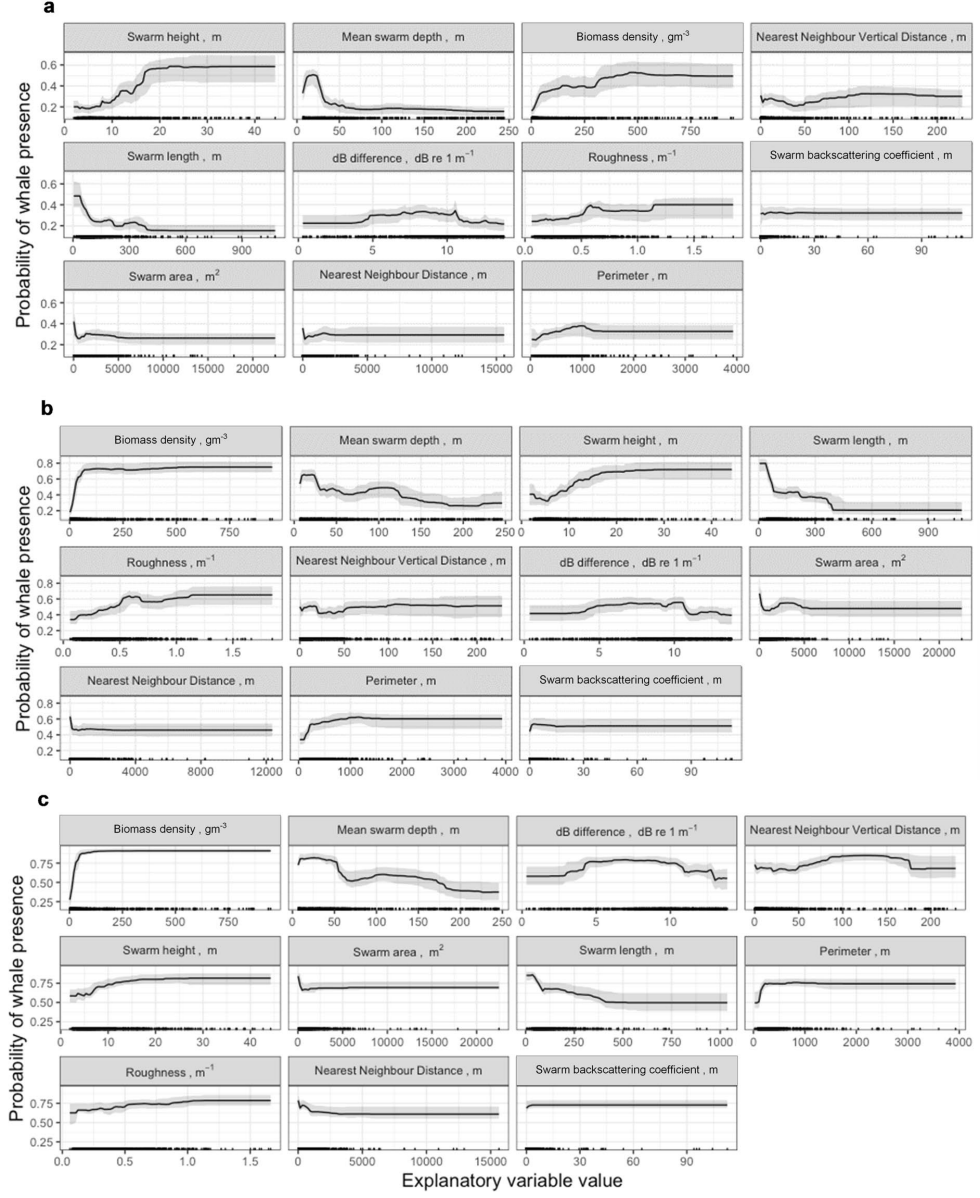
Marginal effects for each explanatory variable in the boosted regression tree (grey shaded area represents 95% CI) for each spatiotemporal scale where whales were classified as ‘present’ if detected within (**a**) 12 km/1 hr, (**b**) 20 km/2 hr, or (**c**) 40 km/4 hr of a krill swarm. The distributions of observed krill swarms are indicated by the carpet plot on each panel.

At the smallest spatiotemporal scale (within 12 km and 1 hour of a whale detection), the contribution of explanatory variables to BRT model fit was greatest for mean swarm height and depth, and density (Table 4; Fig. 4). The results from the two larger spatiotemporal scales showed similar general trends, though krill density was of increasing influence (Table 4; Fig. 4). Overall, the probability of Antarctic blue whale presence increased with increasing numerical density of krill, and krill swarms within the vicinity of blue whales also occurred at shallower depths in the water column (≲30 m) and were greater in height (≳17 m) (Fig. 4). These relationships generally correspond to the patterns of krill swarm properties mapped in Fig. S2 (Supplementary Material) when compared to the distribution of Antarctic blue whales as shown in Fig. 1.

**Table 4:** Relative influence of the explanatory variables in the boosted regression tree models at each spatiotemporal scale where whales were classified as ‘present’ if detected within 12 km/1 hr, 20 km/2 hr, or 40 km/4 hr of a krill swarm. Explanatory variables are ranked in order of influence from high to low.

12 km/1 hour		20 km/2 hours		40 km/4 hours	
Explanatory variable	Percentage influence	Explanatory variable	Percentage influence	Explanatory variable	Percentage influence
Mean height	13.73	Biomass density (wet-weight)	21.64	Biomass density (wet-weight)	28.12
Mean depth	13.67	Mean depth	10.98	Mean depth	12.64
Biomass density (wet-weight)	13.20	Mean height	10.70	dB difference	9.62
Nearest neighbour depth	10.42	Corrected length	9.94	Nearest neighbour depth	8.11
Corrected length	10.34	Roughness	8.26	Mean height	7.53
dB difference	8.17	Nearest neighbour depth	7.5	Corrected area	7.18
Roughness	6.75	dB difference	7.42	Corrected length	7.03
Swarm backscattering coefficient	6.42	Corrected area	6.98	Corrected perimeter	5.58
Corrected area	5.90	Nearest neighbour distance	5.93	Roughness	5.58
Nearest neighbour distance	5.78	Corrected perimeter	5.93	Nearest neighbour distance	5.08
Corrected perimeter	5.62	Swarm backscattering coefficient	4.26	Swarm backscattering coefficient	3.73

**Figure 5:**
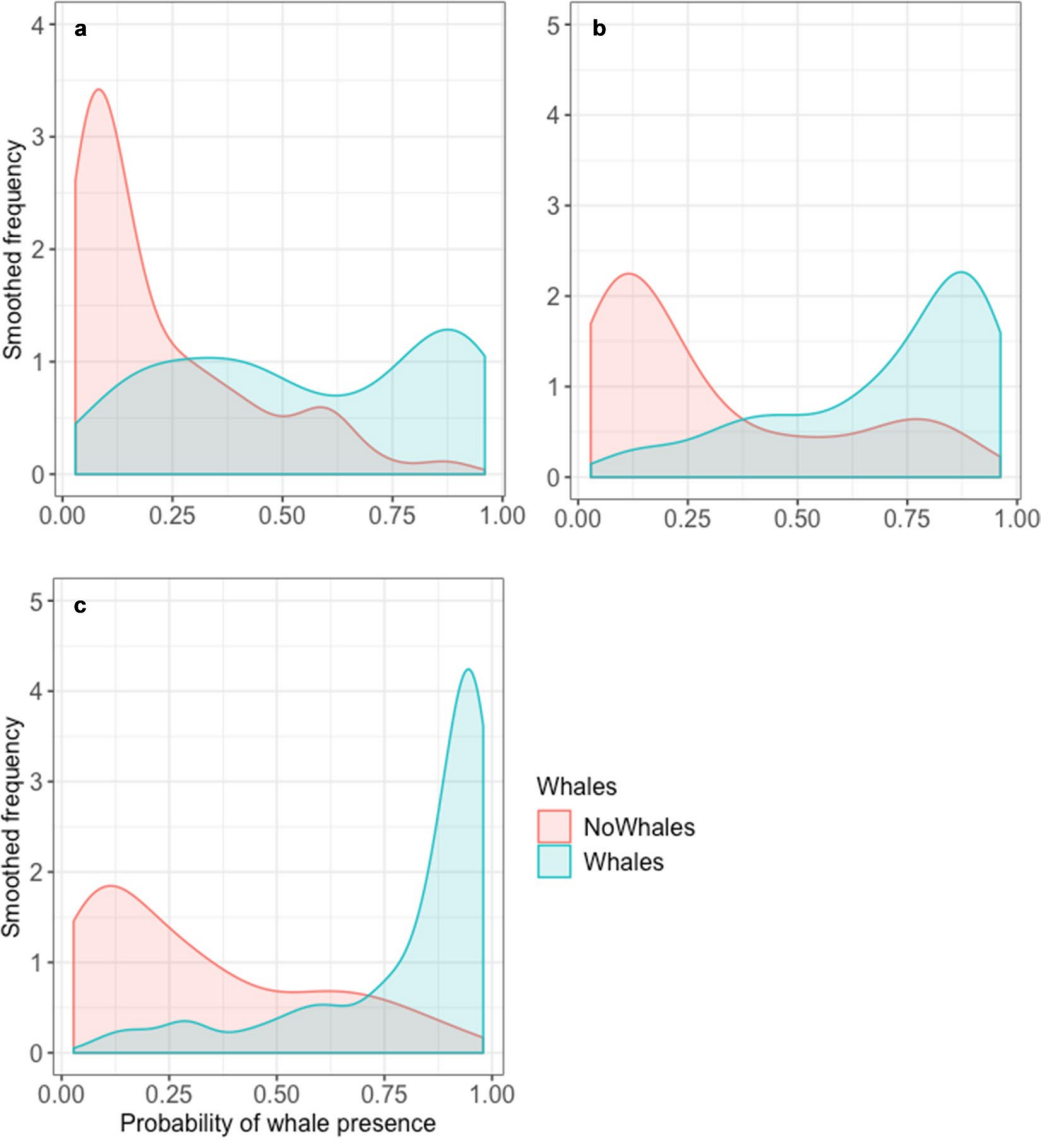
Predictive performance of the boosted regression tree models for each spatiotemporal scale: (**a**) 12 km/1 hr; (**b**) 20 km/2 hr; (**c**) 40 km/4 hr, evaluated using 25% of the total observed krill swarms retained for model testing (N = 268 swarms). The x-axis is the predicted probability of whale presence for each krill swarm detected, grouped according to whether the krill swarm was actually observed with whales (shaded blue) or not (shaded red). The y-axis is the smoothed frequency of observations.

Predictive performance of the models was very good and increased with increasing scale (12 km/1 hour: AUC = 0.83; 20 km/2 hours: AUC = 0.85; 40 km/4 hours: AUC = 0.91). At the smallest scale, the model could more accurately predict when blue whales were absent than when they were present, but the models for both larger scales could readily distinguish when whales were present or absent (Fig. 5). For the largest scale, whale presence was more accurately predicted than whale absence (Fig. 5).

## Discussion

The characteristics of krill swarms observed throughout the survey where highly variable (Table 2). For example, they ranged from mean depths of 7 to 248 m, nearest neighbour distances of <1 m to 23 km, and densities of 2 to 1725 gm^−3^. Despite the overall variability, some characteristics of krill swarms appeared to show spatial structure throughout the survey area (Fig. S2, Supplementary material). Perhaps not coincidentally, this spatial structure appeared broadly related to the distribution of blue whales (Fig. 1). As with previous surveys within this region^39,40^, our survey observed that Antarctic blue whales formed persistent aggregations, supporting previous hypotheses that they have a patchy distribution^42,43^. Our ability to conduct an ecological study within the vicinity of these rare whales was greatly facilitated by recent advances in passive acoustic technology, which allowed efficient detection and tracking of blue whales from over 200 km away^39^.

The results from our models suggest that the numerical density of krill and the depth and height of krill swarms were the most important characteristics for predicting the presence of blue whales (Table 4). When krill swarms were dense (≳300 g m^−3^), shallow (≲30 m depth) and tall (≳15 m height), Antarctic blue whales were likely to be present in close proximity or within at least 40 km of the krill swarm (Fig. 4). The relationship between blue whale presence and krill density was strong at all scales, increasing in importance at broader spatiotemporal scales (Table 4). The predictive performance of our boosted regression tree models was very good, meaning that our ability to predict whale presence around a krill swarm based on its intrinsic characteristics was high (Fig. 5). This performance was limited by sample size, however. For example, our ability to correctly distinguish whale presence improved with increasing spatiotemporal scale (Fig. 5), as the ratio of whale presence to absence for our observed krill swarms increased (Table 5).

**Table 5:** Sample sizes (number of krill swarms) for the training and testing data used to develop the boosted regression trees, and test their predictive performance, respectively. Presence indicates an Antarctic blue whale detection within the associated spatiotemporal scale of the krill swarm detection.

Spatiotemporal scale	Dataset	Blue whale presence	Blue whale absence	Total	Ratio of blue whale presence/absence
12 km/1 hr	Training	280	524	804	0.54
	Test	94	174	268	0.54
20 km/2 hr	Training	396	408	804	0.97
	Test	132	136	268	0.97
40 km/4 hr	Training	497	307	804	1.62
	Test	166	102	268	1.62

The use of passive acoustics allowed us to detect and localise blue whales not only during the day, but also at night. Despite this, the amount of whale search effort, and subsequently the number of krill swarms included in our analysis, were considerably lower during hours of darkness (Table 3; Fig. 3). This is due to both the more limited whale search effort at night than during the day, and the short nights during Antarctic summer. The numbers of krill swarms detected in the presence and absence of whales were reasonably well balanced during daylight hours, however the majority of swarms detected at night were in the presence of whales (Table 3; Fig. 3). This is an artefact of our data collection, as passive acoustic whale effort was typically only conducted at night when whales were thought to be nearby, in an effort to conserve our limited number of sonobuoys. Given that the majority of krill swarms included in our analysis were detected during the day however, this night-time imbalance in sampling with respect to whale proximity is unlikely to be driving the trends in our BRT model.

Our results suggest that the form of krill associated with Antarctic blue whales appears to be neither widely nor evenly distributed throughout our study area – at least during the time of our surveys. Inspection of the spatial distribution of these krill swarm properties reveals relatively strong geographic stratification (Fig. S2, Supplementary Material). We suggest that these patterns are almost certainly driven by some combination of the environmental conditions and the life history of krill. Future studies investigating the physical and biological environment in the vicinity of krill swarms could provide further understanding of this apparent stratification, as well as insight into how blue whales find these dense, shallow krill swarms.

As such swarms are not randomly distributed in space, this suggests that blue whales are actively targeting these swarms as it is energetically advantageous to do so. Goldbogen *et al*.^44^ hypothesised that Antarctic blue whales prefer to feed on shallow, high density krill swarms in order to maximise their energy intake per unit effort, and that diffuse krill layers are unlikely to sustain them. Rorqual whales (Balaenopteridae) feed by engulfing discrete, high volumes of prey-laden water during high velocity lunges^2^. Compared with the continuous filter feeding of right whales (Balaenidae), lunge feeding is energetically expensive. This is particularly the case for larger whales, which limits foraging time and dive time^3^. This suggests that it would be energetically optimal for large lunge feeders such as blue whales, to target high density, large aggregations of shallow krill swarms, while smaller species may be less restricted by depth and search time.

Although we have quantified the difference in krill swarm characteristics in the vicinity of Antarctic blue whales, we cannot conclusively determine cause or effect with the current available data. While blue whales may be targeting shallow, high density krill swarms, these swarm characteristics could to some degree be influenced by whale presence. It was also beyond the scope of this study to consider other factors (e.g., proximity to the ice edge) that may influence the distribution and characteristics of krill swarms. To investigate these questions in future surveys, spatially structured transect designs could be used to examine krill swarms in relation to the surrounding environment and the use of a multibeam would allow for greater coverage and three-dimensional observations of the shape and surface area of entire krill swarms.

Our results share similarities with studies of other Antarctic baleen whales and krill on the summer feeding grounds. As with the blue whales in this study, humpback whales in the Antarctic have been found to be more associated with shallow krill swarms^29^. The biomass of Antarctic krill has been found to have a positive spatial association with both fin^26^ and humpback whales^29^. South Georgia is known to be an important feeding ground for southern right whales, and recent findings suggest that the reproductive success of this population is directly influenced by krill availability, as indicated by a positive correlation in krill densities and the number of calves sighted during the subsequent breeding season^45,46^. The link between the number and characteristics of krill swarms and overall krill biomass^47^ has yet to be explored in the East Antarctic but could provide further information on the availability of krill to blue whales.

Comparing whale sightings to krill length frequencies observed using nets, Santora *et al*.^25^ found different whale species were associated with specific length ranges of krill, with humpbacks showing preference for juvenile krill, fin whales feeding on mature krill and minke whales intermediate to these two. Here, we find some evidence that blue whales are more often associated with medium-sized to mature krill (dB difference: 5 to 10 dB re m^−1^). This result suggests that blue whales may be targeting krill with higher energy content, or that these krill are more readily available to blue whales in this region, perhaps in greater abundance or density.

With the available data from our study we are not able to definitively conclude that all whales detected within the vicinity of krill were indeed feeding. However, whales were at times observed surface feeding, and a small number of video-tracked focal follows of whales at the surface show diving and movement behaviour that suggests foraging^48^. Additionally, blue whale frequency-modulated d-calls^49^, which have been found to be associated with social behaviour and possibly foraging^50^, were detected frequently while surveying the blue whale aggregation^48^. Future investigation of our dataset could specifically examine patterns in the detection of d-calls in relation to krill swarm distribution and characteristics.

The only other study to focus on correlations between Antarctic blue whales and krill, found a negative correlation between blue whale calls and krill biomass^34^. While this may appear to counter our results, their study had several fundamental differences, in that it took place off the Western Antarctic Peninsula outside of the summer feeding season, and was part of a broader, spatially structured, oceanographic survey with no dedicated ship time to track down whales. No blue whales were sighted during their survey and they had no means of measuring distances to acoustic detections. As acoustic measurments of krill are made directly below the ship, there can be major spatial mismatch if detected whales are far from this location, particularly given blue whales can be heard from hundreds of kilometers away^39,51,52^. By tracking down blue whales and measuring distance both visually and acoustically, we were able to compare the characteristics of krill swarms within the vicinity of Antarctic blue whales to those demonstrably far away.

Overall, our study has provided new insights into the sub meso-scale (i.e., <100 km) foraging behaviour of Antarctic blue whales, and has demonstrated that the combination of visual observations and recent advances in passive acoustic methods provide efficient and robust means of undertaking ecological studies in the vicinity of these rare whales in a challenging environment. Additional research using state-of-the-art active acoustic technology and further integration of environmental, oceanographic and biogeochemical data, would expand upon this analysis and allow observation of fine-scale interactions encompassing not only predator-prey, but ecosystem-wide relationships. As blue whales and krill are both ‘ecosystem engineers’ of the Southern Ocean^36–38^, understanding the nature of their predator-prey dynamics in a changing environment is important; not only for the recovery of this endangered species, but also for the management of the Antarctic krill fisheries and the Antarctic ecosystem as a whole^6,53^. Further knowledge of the foraging requirements of top Antarctic predators and the level of niche overlap between them and the krill fishery will be of increasing importance for future environmental monitoring. Given the level of natural variability in the marine environment, the increasing impacts of climate change and fishing pressure may lead to greater interspecific competition for shared and limited resources.

## Methods

### Data collection

The multidisciplinary research voyage was conducted from the RV *Tangaroa*, operated by New Zealand’s National Institute of Water and Atmospheric Research Limited (NIWA). The voyage lasted 42 days, departing from Wellington, New Zealand on January 29^th^, 2015 and returning to the same port on 11^th^ March 2015. During the voyage we had 13.5 days of ship time to conduct dedicated blue whale research, from February 8^th^ to 14^th^ and February 24^th^ to March 2^nd^.

Following the methods described in Miller *et al*.^39^, DIFAR sonobuoys were deployed at 55 km (30 nmi) or 3 hour intervals, or adaptively when needed, throughout both daylight and night-time hours, and bearings were used to guide the ship towards groups of vocalising Antarctic blue whales. The proximity to blue whales was estimated in real-time based on the intensity of vocalisations, range of bearings, and changes in bearings from the series of individually monitored sonobuoys. To more precisely determine the location of vocalising whales thought to be nearby, acousticians deployed and concurrently monitored two sonobuoys to obtain cross-bearings (i.e. triangulations). All passive acoustic data were analysed using the DIFAR module in PAMGuard^54^. Three categories of blue whale vocalisations were detected: unit ‘a’ calls (single unit tonal), full ‘z’ calls (3-unit ‘song’), and frequency-modulated d-calls^55^.

Visual observations of whales were conducted continuously throughout the voyage during all daylight hours, when weather permitted. A minimum of two observers were on-watch from the open-air flying bridge or enclosed bridge depending on weather conditions. Observers alternated between searching with 7x binoculars and the naked eye. For each cetacean sighting, the distance and angle relative to the ship’s course were estimated using reticle binoculars and mounted angle boards; some sighting distances close to the vessel were estimated by observers without the use of reticles. When weather permitted, sightings and acoustic detections of whales thought to be blue whales were investigated to obtain visual confirmation of the species, estimate group size, obtain photographic identification, biopsies, and conduct focal (i.e. behavioural) follows.

The ability to track the location of blue whales in real-time facilitated the collection of active acoustic data in their vicinity and within regions demonstrably far from any vocalising or surfacing blue whales. Active acoustic data were obtained continuously using a calibrated scientific echosounder (Simrad EK60, Horten, Norway). The echosounder operated at 38 and 120 kHz for the duration of the voyage with a pulse duration of 1.024 ms, a pulse repetition rate of one ping per second and a 7° beam width. Acoustic data were processed using Echoview v6.1 (Myriax, Hobart, Australia) and the R package EchoviewR^56^. Background and time varied gain noise was removed using the method outlined in De Robertis and Higginbottom^57^. Surface noise, seabed and seabed alias echoes were also removed prior to delineation of aggregations.

A 7 × 7 convolution filter was applied to the 38 and 120 kHz clean echosounder data from below the surface exclusion (mean depth = 10 m) to a maximum depth of 250 m. The shoal analysis and patch estimation system (SHAPES^58^) algorithm implemented in Echoview was run on the 120 kHz echosounder data using parameters validated in previous krill studies^8,9^. Krill length frequency distribution (Fig. 2) was determined using targeted tows with a fine-mesh midwater trawl that had a circular opening of 12 m diameter and a codend mesh of 10 mm. During target fishing the net was towed for 20–30 min at 3–4 knots. Trawl data, depth, door spread and headline height were obtained using a Furuno CN22 net monitor. The morphology of resulting aggregations were corrected for echosounder beam characteristics using the methods of Diner^59^.

Aggregations were identified as krill using a dual frequency ‘dB-difference’ technique where 120–38 kHz mean volume backscattering strength (MVBS)^60^ was calculated for each aggregation^8,9^. Krill acoustic target strength (TS) was calculated using the model of Calise and Skaret^61^. In the TS model, krill length is determined by trawling with all other model parameters held fixed at the settings of Calise and Skaret^61^. Aggregations with a dB-difference (120–38 kHz) falling between 1.04 to 14.80 dB re 1 m^−1^ were identified as krill.

Krill swarms were characterised using 11 variables (see Results: Table 2). Biomass density (wet-weight), *ρ*_*v*_ was calculated using *ρ*_*v*_ = ${10}^{\{(MBV{S}_{120}-T{S}_{kg})/10\}}$ where *TS*_*kg*_ is the target strength of 1 kg of krill at 120 kHz using the length to wet-mass relationship of Morris *et al*.^62^.

### Krill swarm classification

Frequent changes in the ship’s bearing are likely to impact the accuracy of characterising krill swarms so those detected while the ship’s heading was highly variable were removed from analyses. This was done by a simple visual assessment of the ship’s track. Krill were classified as being in the presence of Antarctic blue whales based on the spatiotemporal distance to the nearest whale detection (visual sighting and/or passive acoustic triangulation). Thus, for the purpose of this study we define ‘whale effort’ as the time periods when we could measure the distance to blue whales (relatively) precisely. Whale visual sighting effort was defined as periods when observers were on search duty from the flying or enclosed bridges. Passive acoustic effort was defined as periods when two sonobuoys were deployed simultaneously so that it was possible to triangulate locations and estimate distances to calling whales. The inclusion of passive acoustic whale effort allowed us to measure distances to whales not only during the day but also at night. Krill swarms detected outside periods of whale effort (either visual sightings or passive acoustic triangulation) were excluded from analyses, since there was lower certainty regarding the distance of whales to these swarms.

Initially, all krill swarms detected within 12 km and 1 hour of whale detections were classified as being in the presence of whales for this analysis. To assess how the relationships between krill swarm characteristics and whale presence changed at different spatiotemporal scales, krill swarms were additionally reclassified as being in the presence of whales based on two other thresholds (within 20 km and 2 hours of a whale detection, and within 40 km and 4 hours of a whale detection). These scales were chosen to reconcile the different scales of our observation systems. Blue whales could be visually sighted up to 12 km from the ship and acoustically triangulated out to ~40 km, whereas krill were observed directly below the ship.

### Statistical analysis

Krill swarm characteristics in relation to whale presence/absence (*y* = 1 for presence) were modelled using Boosted regression trees (BRTs)^63,64^ via a logit: *logit*(P(y = 1|**X**)) = *f*(**X**) where **X** is the 11 measured characteristics for each krill swarm. All statistical analyses were carried out using R^65^ version 3.4.2 and the gbm R package version 2.1.3^66^.

The krill swarm dataset contained extreme outliers that are problematic during modelling, so for the *j*th covariate, the *i*th observation was removed when *X*_*ij*_ > *Q*_0.995_ (X_*j*_), where *Q*_0.995_ is the 99.5% quantile. The krill observations were then randomly allocated to either the training dataset (75%, N = 804 swarms) for use during model fitting, or the testing dataset (25%, N = 268 swarms) used for the assessment of model performance via proportional stratification, such that the ratio of whale presence/absence observations was kept constant between the two datasets (Table 5).

BRTs achieve local regularisation, and prevent overfitting, by jointly optimising the number of trees (nt), learning rate (lr), and tree complexity (tc)^63^. Model optimisation was carried out by minimising deviance during a grid search (i.e. all possible combinations) of nt, lr and tc = using the R package ‘caret’^67^ and the following settings: lr = {0.1, 0.05, 0.01, 0.005. 0.001, 5 × 10^−4^}, nt = {100, 600, 1100, …, 10000}, and tc = {1, 2, 3, 5, 7, 10}. A bag fraction of 0.5, or 50% of the training data was used during each series of model fits. Ten-fold cross validation was used to estimate the best performing model, i.e. the model with the lowest deviance, for a given combination of nt, lr, and tc.

The area under the receiver operating characteristic curve (AUC) was used to assess the discriminatory ability of the model^68^. AUC values of 0.5 represent models not able to discriminate between krill swarms without whales and krill swarms with whales, and values nearer 1 represent models with very good discriminatory ability.

## Supplementary information

Dataset 1

Supplementary Material: The characteristics of krill swarms in relation to aggregating Antarctic blue whales

## Data Availability

Data used in this study are publicly available as follows: Whale passive acoustic and visual sightings data are available through the Australian Antarctic Data Centre: https://data.aad.gov.au/metadata/records/AAS_4102_2015_New_Zealand_Australia_Antarctic_Ecosystems_Voyage (Andrews-Goff *et al*. 2017). Krill acoustic data used in our analysis are available in the Supplementary Material.
