# Supplementary material for: The characteristics of krill swarms in relation to aggregating Antarctic blue whales
Source: Sci Rep. 2019 Nov 11;9:16487. doi: 10.1038/s41598-019-52792-4 (PMC6848198; doi:10.1038/s41598-019-52792-4)
Supplement: Supplementary file 2 — Supplementary Material: The characteristics of krill swarms in relation to aggregating Antarctic blue whales [file 41598_2019_52792_MOESM2_ESM.docx]

# **Supplementary Material**

The characteristics of krill swarms in relation to aggregating Antarctic blue whales

Miller, E. J.^1,2,*^, Potts, J. M.^3^, Cox, M. J.^1^, Miller, B. S.^1^, Calderan, S.^4^, Leaper, R.^5^, Olson, P. ^6^, O’Driscoll, R. L. ^7^, Double, M. C.^1^

^1^ Australian Antarctic Division, 203 Channel Highway, Kingston, Australia

^2^ E Miller Consulting, Hobart, Tasmania, Australia

^3^ The Analytical Edge, PO Box 47, Blackmans Bay, Tasmania, Australia

^4^ Scottish Association for Marine Science, University of the Highlands and Islands, Oban, Argyll, UK

^5^ International Fund for Animal Welfare, 87-90 Albert Embankment, Lambeth, London, UK

^6^ Southwest Fisheries Science Center, National Marine Fisheries Service/National Oceanic and Atmospheric Administration, La Jolla, California, USA

^7^ National Institute of Water & Atmospheric Research Limited, Wellington, New Zealand

*Corresponding author: [*elanorjh@gmail.com*](mailto:elanorjh@gmail.com)


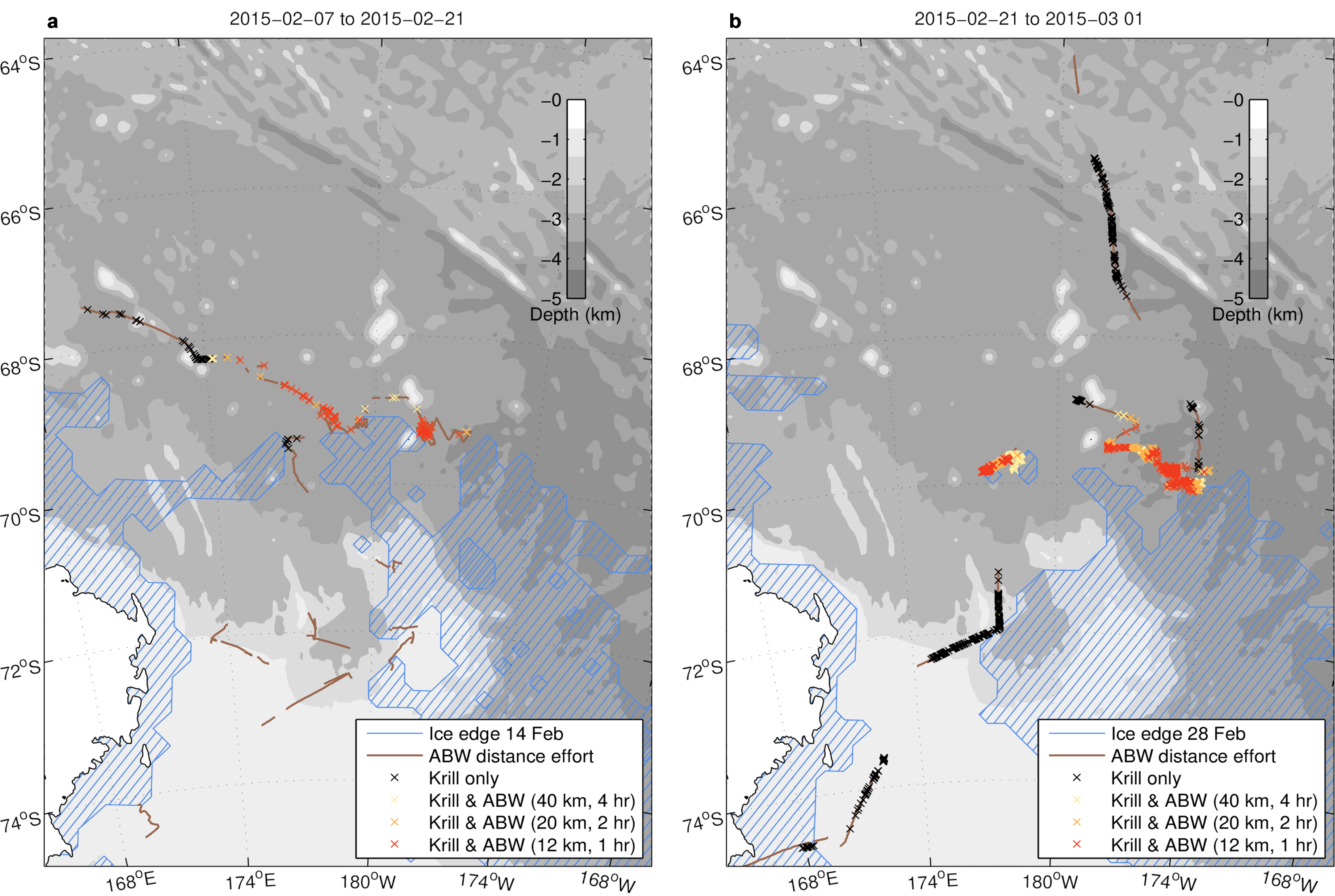


**Figure S1.** Locations of krill swarms detected in the Ross Sea survey area. Colours indicate the most proximate distance & timespan for which whales were considered present. Brown line indicates effort for distance measurement of whales (visual or either passive acoustic). **a)** Survey data from 07-21 Feb 2015 with ice edge shown for 14 Feb. **b**) Survey data from 21 Feb – 01 Mar with ice edge shown for 28 Feb.



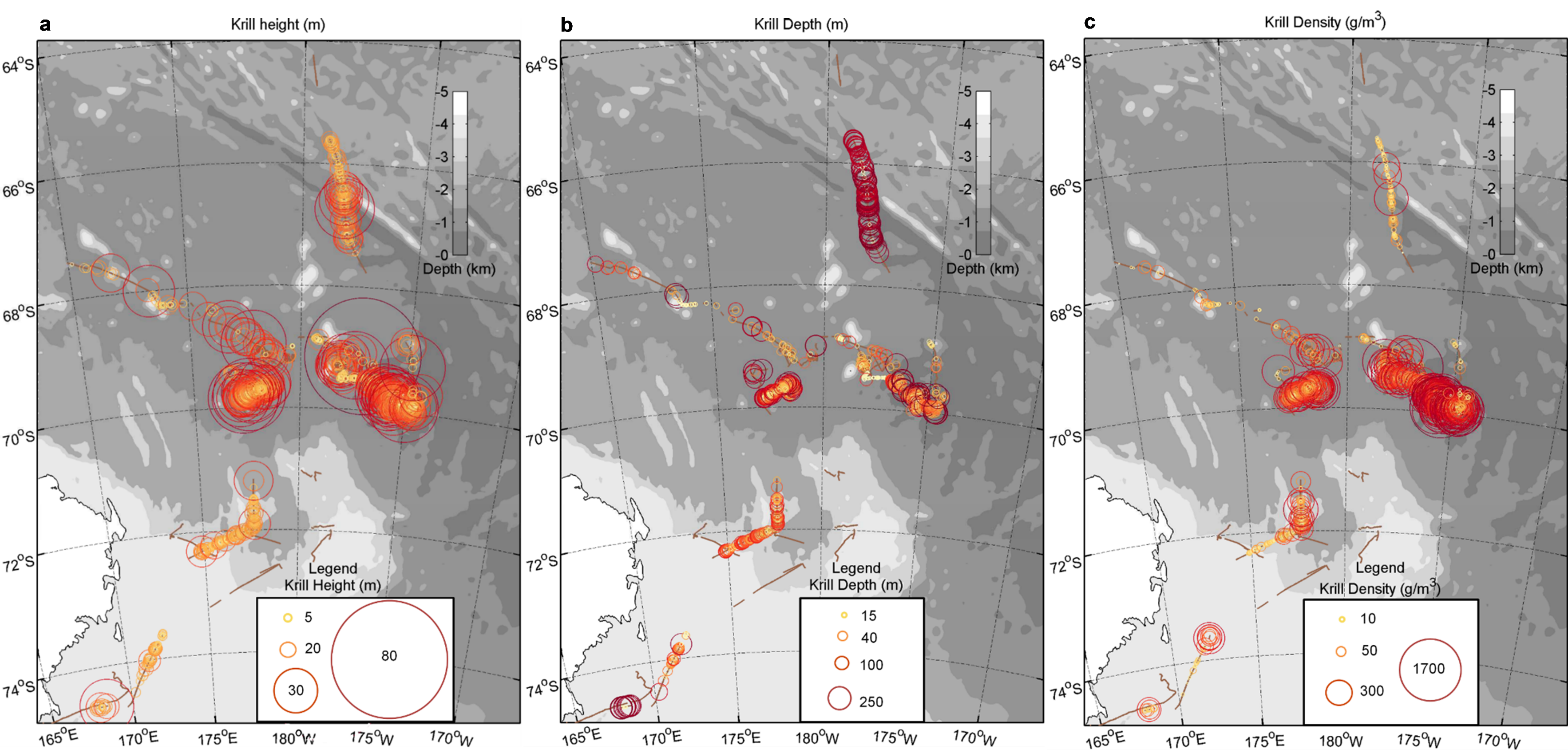



**Figure S2***:* Properties of krill swarms detected throughout the survey region during periods of effort for whale localisation (visual or passive acoustic). **a**) Krill swarm mean height. **b**) Krill swarm mean depth in the water column. **c)** Krill density in g/m^3^.
